# High-dose romiplostim for donor-type aplasia

**DOI:** 10.1007/s00277-025-06448-1

**Published:** 2025-06-12

**Authors:** Kohei Hosokawa, Tatsuya Imi, Takafumi Yokota, Yuma Tada, Hiroyuki Maruyama, Naomi Sugimori, Ken Ishiyama, Hirohito Yamazaki, Toshihiro Miyamoto, Shinji Nakao

**Affiliations:** 1 Department of Hematology, Faculty of Medicine, Institute of Medical Pharmaceutical and Health Sciences, Kanazawa University, 13-1 Takaramachi, Kanazawa, 920-8641 Ishikawa Japan; 2 Department of Hematology, Osaka International Cancer Institute, Osaka, Japan; 3 Department of Hematology and Oncology, Graduate School of Medicine, Osaka University, Suita, Osaka Japan; 4 Division of Transfusion Medicine, Kanazawa University Hospital, Kanazawa, Japan; 5 Japanese Red Cross Ishikawa Blood Center, 4-445 Fujiekita, Kanazawa, 920-0345 Ishikawa Japan

**Keywords:** Romiplostim, Donor-type aplasia, Allo-BMT

## Abstract

Donor-type aplasia (DTA) is a severe complication of allogeneic hematopoietic stem cell transplantation (allo-HSCT), characterized by bone marrow hypoplasia despite full chimerism. This report highlights the successful use of romiplostim (ROMI) for treating DTA in three patients with acquired aplastic anemia (AA), including two who were unresponsive to eltrombopag (EPAG). Case 1: A 21-year-old female with non-severe AA, treated with cyclosporine (CsA), rabbit antithymocyte globulin, and EPAG, showed no improvement. After undergoing bone marrow transplantation (BMT) from an HLA-matched sibling, she continued to experience pancytopenia. Switching from EPAG to ROMI 16 months after BMT led to transfusion independence after 7 weeks and normalized blood counts by 17 months. Case 2: A 35-year-old male with moderate AA, initially treated with CsA and ROMI, switched to EPAG before BMT from an HLA-matched sibling. Despite complete donor chimerism, pancytopenia recurred 7 months after BMT. Transitioning from EPAG to ROMI resulted in transfusion independence after 5 months and normalized blood counts after 11 months of ROMI. Case 3: A 25-year-old female with moderate AA, unresponsive to CsA and EPAG, remained transfusion-dependent. Following BMT from an HLA-matched sibling and an initial lack of response to ROMI, restarting ROMI led to transfusion independence after 9 months and normalization of blood counts by day 418 post-BMT. These patients suggest ROMI, particularly at high doses, may be more effective than EPAG for DTA, potentially avoiding the need for a second allo-HSCT. Further studies are required to compare the efficacy of ROMI and EPAG in treating DTA following allo-HSCT.

## Background

Donor-type aplasia (DTA) is a life-threatening complication of allogeneic hematopoietic stem cell transplantation (allo-HSCT) characterized by bone marrow (BM) hypoplasia with full chimerism. The incidence of DTA after allo-HSCT for acquired aplastic anemia (AA) is relatively low among Caucasians [1]. In a nationwide registry of pediatric patients with AA in Japan, DTA occurred in 5.7% of cases overall, and in 11% of those who received Fludarabine-based conditioning [2]. An immune attack against hematopoietic stem cells (HSCs) is involved in approximately 50% of patients [3, 4]. Various treatments, including immunosuppressive therapy and boost infusion of HSCs, have been attempted. Some patients may respond to a thrombopoietin receptor agonist (TPO-RA) eltrompopag (EPAG) [5–7]. However, these treatments have limited effect, and many patients eventually require a second allo-HSCT. We report the successful treatment of DTA in three AA patients treated with romiplostim (ROMI), including two patients who did not respond to EPAG. All patients provided their informed consent in accordance with the principles of the Declaration of Helsinki.

## Case presentation

**Fig. 1:**
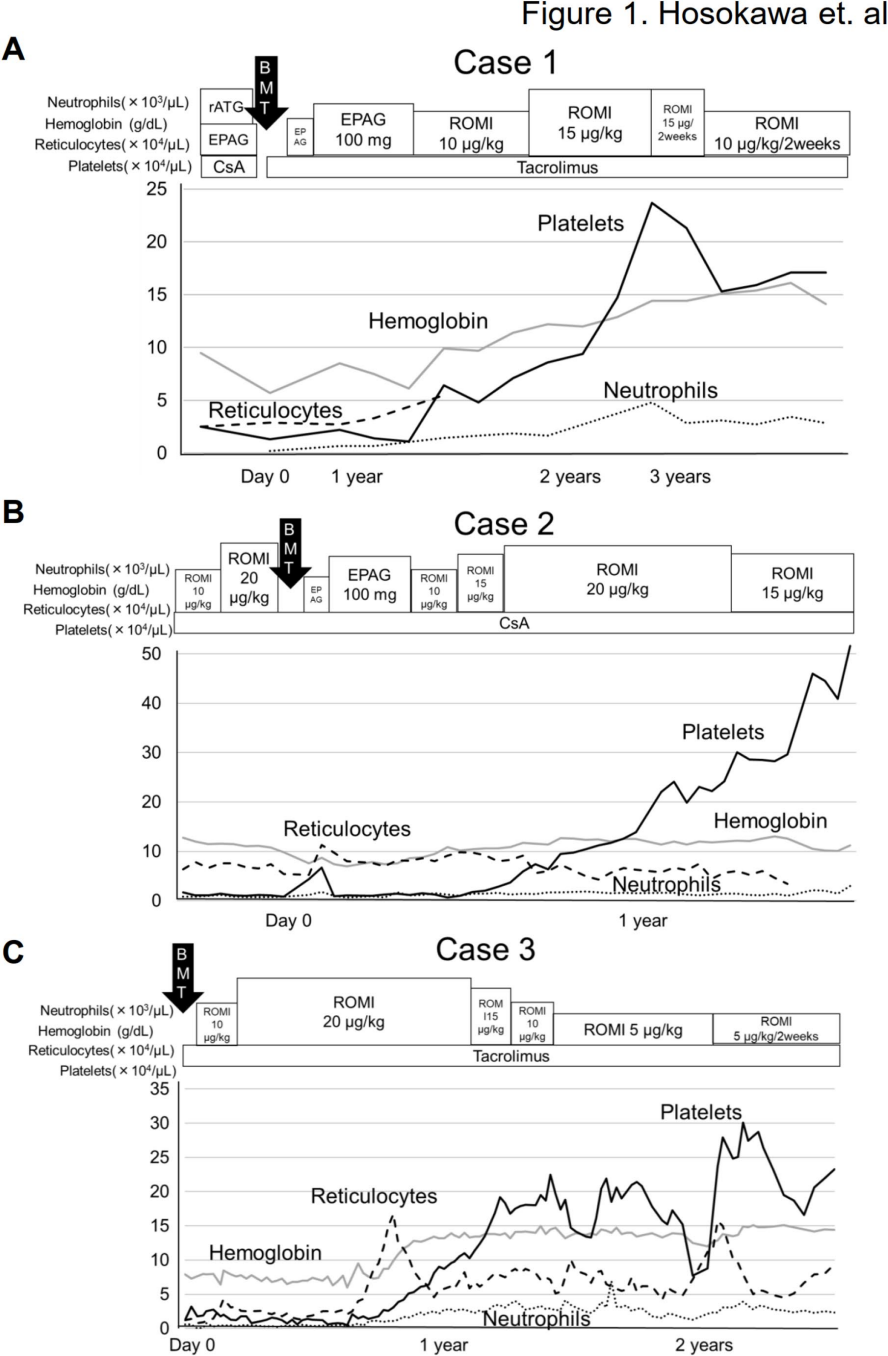
Successful treatment of donor-type aplasia with romiplostim in three acquired aplastic anemia patients, including two patients who did not respond to EPAG. **A** The clinical course of Case (1) EPAG was switched to ROMI (10 µg/kg/week) 16 months after BMT. Her pancytopenia started to improve after 7 weeks of ROMI therapy, and she became transfusion independent 3 months later. **B** The clinical course of Case (2) EPAG was changed to ROMI starting at 10 µg/kg/week and was kept at the dose of 20 µg/kg for 5 months. Five months after the initiation of ROMI therapy, he achieved transfusion independence, and his blood cell counts normalized 11 months after the therapy. **C** The clinical course of Case (3) We diagnosed the patient as having DTA, and ROMI (10 µg/kg/week) was restarted on day 43, and increased up to 20 µg/kg/week. Seven months after BMT, her reticulocyte counts increased from 21.0 × 10^9^/L to 40.0 × 10^9^/L, and she achieved transfusion independence 9 months after BMT

The first patient (**Case 1**), a 21-year-old female, was diagnosed with non-severe AA at 17 years of age and was observed without treatment for 4 years. As pancytopenia gradually progressed, she received cyclosporine (CsA) monotherapy followed by a combination of rabbit antithymocyte globulin (ATG), CsA, and EPAG, without any response. She underwent BMT from an HLA-matched male sibling donor five years after the initial diagnosis. The total number of nucleated cells was 2.05 × 10^8^/kg. The conditioning regimen included fludarabine, melphalan, and total body irradiation (TBI) (4 Gy at Day − 1). Tacrolimus and short-term methotrexate (sMTX) were administered as GVHD prophylaxis. Neutrophil engraftment was observed on day 18. However, all blood cell counts started to decrease from day 55. A fluorescent in situ hybridization (FISH) analysis revealed complete donor chimerism. EPAG was restarted 7 months after BMT and continued for 8 months at a dose of 100 mg/day, the maximum dose approved in Japan. Although her hemoglobin and platelet counts slightly increased from 5.7 to 7.5 g/dL and 13.0 × 10^9^/L to 14.0 × 10^9^/L, respectively, after EPAG, she remained transfusion-dependent. Therefore, a second BMT from the same donor was considered. However, as the patient declined the second BMT, we switched from EPAG to ROMI (10 µg/kg/week) 16 months after BMT. Her pancytopenia started to improve 7 weeks after ROMI, and she became transfusion-independent 3 months later (Fig. 1A). After continuing ROMI (15 µg/kg/week) for 17 months, her blood cell count completely normalized. ROMI was tapered to 5 µg/kg once every 2 or 3 weeks at 3 years and discontinued at 5 years after BMT without recurrence of pancytopenia. No adverse events associated with ROMI were observed.

The second patient (**Case 2**), a 35-year-old male, was diagnosed with moderate AA at 32 years of age. He was treated with CsA and ROMI for 7 months; however, he did not respond and became platelet transfusion-dependent. ROMI was switched to EPAG, which was continued for 2 months without any effects. He underwent BMT from an HLA-matched, ABO-major incompatible female sibling donor. The total number of nucleated cells after erythrocyte removal was unexpectedly low (0.7 × 10^8^/kg). The conditioning regimen included fludarabine, cyclophosphamide (25 mg/kg from days − 6 to −3), and rabbit ATG (1.25 mg/kg on days − 4 and − 3). CsA and sMTX were used as GVHD prophylaxis. Neutrophil engraftment occurred on day 17, but no signs of erythrocyte or platelet engraftment were observed on day 32. A FISH analysis showed that 11.6% of the peripheral blood leukocytes were of recipient origin. EPAG was started on day 34 and continued at a dose of 100 mg/day. After achieving complete donor chimerism 4 months after BMT, he transiently became transfusion-independent (WBC, 4.49 × 10^9^/L; hemoglobin, 8.9 g/dL; platelet count, 39.0 × 10^9^/L). However, pancytopenia recurred, and he became transfusion-dependent again seven months after BMT. We changed the treatment from EPAG to ROMI starting at 10 µg/kg/week and maintained it at a dose of 20 µg/kg for 5 months. At five months after the initiation of ROMI, the patient achieved transfusion independence, and his blood cell counts normalized 11 months after ROMI (Fig. 1B). However, his pancytopenia recurred after prolonged treatment with ibrutinib for chronic GVHD despite the continuous administration of ROMI (20 µg/kg) and he eventually died of severe intestinal GVHD on day 962 after BMT.

A female patient (**Case 3**) was diagnosed with moderate AA at 25 years of age. Although she had idiopathic pulmonary arterial hypertension, there had no physical features or family history suggestive of inherited BM failure syndrome. Telomere length was assessed using flow-FISH at diagnosis and found to be within the normal range for age, making clinically significant telomeropathy unlikely. She was treated with CsA and EPAG but did not respond and eventually became platelet transfusion-dependent. EPAG (100 mg/day) was switched to ROMI at a dose of 10 µg/kg/week and increased to 20 µg/kg/week; however, she showed no response after 3 months. She underwent BMT from an HLA-matched female sibling donor 2 years after her diagnosis. The total number of nucleated cells was 2.56 × 10^8^/kg. The conditioning regimen included fludarabine, melphalan, and rabbit ATG (2.5 mg/kg on days − 6 and − 5). Tacrolimus and sMTX were administered as GVHD prophylaxis. Neutrophil engraftment occurred on day 20, with complete donor chimerism. However, she did not achieve erythrocyte or platelet engraftment and remained transfusion-dependent on day 35. We diagnosed the patient with DTA, and ROMI (10 µg/kg/week) was restarted on day 43 and increased to 20 µg/kg/week. Seven months after BMT, her reticulocyte count increased from 21.0 × 10^9^/L to 40.0 × 10^9^/L, and she achieved transfusion independence 9 months after BMT (Fig. 1C). As all blood cell counts normalized on day 418, ROMI was tapered to 5 µg/kg every 2 weeks at 14 months after BMT, without recurrence of pancytopenia. No severe side effects of the ROMI were observed.

## Discussion and conclusions

Two of the three patients with DTA (**Cases 1** and **2**) were refractory to EPAG before receiving ROMI. Although the treatment duration with the maximum dose of ROMI (20 µg/kg/week) ranged from 5 to 17 months, all three DTA cases achieved trilineage complete responses without any serious adverse events. To the best of our knowledge, this is the first case report of successful ROMI therapy for DTA refractory to EPAG after allo-BMT for AA.

A recent clinical trial showed that ROMI was effective in approximately 80% of EPAG-naïve patients with refractory AA when administered at doses up to 20 µg/kg/week [8]. Further, our retrospective analysis revealed that high-dose ROMI (20 µg/kg being the maximum dose approved in Japan) was highly effective in AA refractory to EPAG [9]; 21 (76%) of 28 EPAG-refractory AA patients showed a response in at least one lineage of cells. A similarly high response rate (70%) to ROMI in EPAG-refractory AA cohorts was shown in another study [10]. Although it was reported as a safe and effective therapy for refractory thrombocytopenia after allo-HSCT [6, 7], the role of TPO-RA in the treatment of DTA has not been established. In the second patient, telomere exhaustion due to initial engraftment with a low stem cell dose may have contributed to the development of DTA. Although retrospective testing was not feasible, this hypothesis warrants further investigation. Given the marked improvement in DTA refractory to EPAG, high-dose ROMI may be more effective in restoring the graft function than EPAG up to 100 mg/day, which is equivalent to 200 mg/day in Caucasian patients. Indeed, the use of high-dose ROMI enabled our three patients to avoid a second allo-HSCT, which is associated with high transplant-related mortality. Our results warrant further studies to compare the effectiveness of ROMI with that of EPAG in the treatment of DTA after allo-HSCT.

## Data Availability

No datasets were generated or analysed during the current study.
